# Supplementary material for: Educating the next generation of cancer researchers: Evaluation of a cancer research partnership training program
Source: PLoS One. 2023 Oct 4;18(10):e0286279. doi: 10.1371/journal.pone.0286279 (PMC10550190; doi:10.1371/journal.pone.0286279)
Supplement: S2 Appendix — (DOCX) [file pone.0286279.s002.docx]

S2 Appendix. SCRI Applicant Cancer Knowledge and Research Interest Survey

## Section 1. Cancer Health Disparities

- 1. Over the past three years, my understanding of cancer health disparities has increased.
     1. strongly agree; my understanding has improved a great deal
     2. agree; my understanding has improved somewhat
     3. neutral/no opinion; no change in my understanding
     4. disagree; I do not feel any more knowledgeable about cancer health disparities
     5. strongly disagree; I am even more confused about this topic
  2. Compared to non-Hispanic white men, how likely are Asian American men to be diagnosed with liver cancer?
     1. more likely
     2. just as likely
     3. less likely
     4. don’t know
  3. Compared to non-Hispanic white women, how likely are Hispanic women to be diagnosed with cervical cancer?
     1. more likely
     2. just as likely
     3. less likely
     4. don’t know
  4. Compared to non-Hispanic white women, how likely are African American women to die of colorectal cancer?
     1. more likely
     2. just as likely
     3. less likely
     4. don’t know
  5. Compared to non-Hispanic white men, how likely are African American men to die of prostate cancer?
     1. more likely
     2. just as likely
     3. less likely
     4. don’t know

## Section 2. Cancer Biology

- 1. Over the past three years of training, my understanding of cancer biology and research has increased.
     1. strongly agree: my understanding has improved a great deal
     2. agree: my understanding has improved somewhat
     3. neutral/no opinion/ no change in my understanding
     4. disagree: I do not feel any more knowledgeable about cancer biology
     5. strongly disagree: I am even more confused about this topic
  2. Which of the following is the best definition of cancer?
     1. uncontrolled division of cells in the body
     2. uncontrolled division of cells that can invade other tissues
     3. genetic mutations leading to uncontrolled division of cells
     4. a mass of abnormal cells, all dividing without normal controls
  3. Which of the following is NOT a difference between cancer cells and normal cells?
     1. Cancer cells grow in the absence of signals telling them to grow while normal cells only grow when they receive such signals
     2. Cancer cells grows slower than normal cells
     3. Cancer cells invade into nearby areas while normal cells stop growing when they encounter other cells
     4. Cancer cells spread to other areas of the body while most normal cells do not move around the body
  4. How likely is a man with obesity (body mass index > 27) to be diagnosed with colorectal cancer relative to a man with a lower body mass index?
     1. more likely
     2. just as likely
     3. less likely
     4. don’t know
  5. Which of the following is the current “gold standard” for evaluating the efficacy of novel cancer treatments?
     1. animal studies
     2. phase 1 clinical trials
     3. phase 2 clinical trials
     4. phase 3 clinical trials
     5. case-control studies

## Section 3. Cancer Prevention and Early Diagnosis

3.1. What is the most common risk factor for liver cancer?

- - 1. Lung cancer
    2. Chronic infection with hepatitis B virus and hepatitis C virus
    3. HIV/AIDS
    4. Tobacco use
  1. What is the recommended screening test for lung cancer?
     1. Sigmoidoscopy
     2. Blood test
     3. Low-dose computed tomography (LDCT)
     4. Papanicolaou test
  2. Which of the following is true about colorectal cancer?
     1. A colon polyps can take as many as 10 to 15 years to develop into colon cancer
     2. A person is considered at increased or high risk of colorectal cancer when they have a family history of colorectal cancer
     3. Stool-based tests (e.g., fecal immunochemical test) are less invasive and easier to have done
     4. All of the above
  3. What of the following is NOT a way to reduce the risk of liver cancer?
     1. Get hepatitis B vaccine
     2. Increase alcohol and tobacco use
     3. Receive treatment for hepatitis C virus if infected
     4. Get to and stay at a healthy weight

## Section 4. Interest in Cancer Research

- 1. How interested are you in pursuing a higher degree (Masters or doctorate) in cancer biology or cancer health disparities disciplines?
     1. Not interested at all
     2. Somewhat interested
     3. Very interested
  2. How interested are you in pursuing a career path in cancer biology or cancer health disparities research in academia?
     1. Not interested at all
     2. Somewhat interested
     3. Very interested
  3. How interested are you in pursuing a career path in teaching cancer biology or cancer health disparities research?
     1. Not interested at all
     2. Somewhat interested
     3. Very interested
  4. How interested are you in pursuing a career path in cancer biology or cancer health disparities in the industry?
     1. Not interested at all
     2. Somewhat interested
     3. Very interested

## Section 5. Demographics

- 1. What is/are your area(s) of scientific specialization? (Select up to two)

Public Health

Epidemiology

Social Work

Nutrition

Molecular Biology Cell Biology Genetics

Biochemistry

Neuroscience

Psychology

Medicine

Dentistry

Nursing

Psychiatry

Engineering

Computer Science

Sociology

Teaching/Education Clinical Practice Clinical Research

Other, please describe

- 1. To which gender identity do you most identify?

Cisgender Male Cisgender Female

Transgender Female Transgender Male

Gender non-binary, genderqueer, or gender fluid

Prefer not to answer

Not listed above, specify: ___________

- 1. What is your race? (check all that apply)

White

Black or African American

Asian

American Indian or Alaska Native

Native Hawaiian or other pacific islander

Other, specify: ____________

Prefer not to answer

- 1. What is your ethnicity?

Hispanic non-Hispanic do not wish to respond

- 1. Are you a first-generation college student in your immediate family?

Yes No do not wish to reply

- 1. Do you have any disabilities?

Yes No do not wish to respond

- 1. What is your current academic standing? [select one]

Undergraduate

Working in healthcare, medicine, or science having completed a BA/BS degree

MS level graduate student

Working in healthcare, medicine, or science having completed an MS degree

Doctoral level graduate student, including PhD student, medical/dental student.

Post-doctoral researcher/scientist

Working outside research/academia, completed PhD degree

No longer working in science

Do not wish to respond

Other, please describe

## Section 6. SCRI Application

## (Only to SCRI Applicants Not SCRI Alum)

- 1. In what year did you apply to the SCRI program? (Choose all that apply)

1. 2019
2. 2020
3. 2021
4. 2022
   1. Have you been accepted to similar cancer research training programs since your application to the SCRI program?
5. Yes
6. No

Do you wish to be entered into a drawing for a chance to win one of ten $20 Amazon gift card?

Yes _____ No_____

If “Yes” please provide your email address: __________
